# Development of A Decahedral Nanoenzyme Capable of Overcoming Hypoxia to Facilitate the Iodine-125 Radiosensitization of Esophageal Cancer

**DOI:** 10.3389/fbioe.2021.764531

**Published:** 2021-10-08

**Authors:** Dechao Jiao, Kunpeng Wu, Kaihao Xu, Yiming Liu, Deyao Zhao, Xinwei Han, Ruitai Fan

**Affiliations:** ^1^ Department of Interventional Radiology, First Affiliated Hospital of Zhengzhou University, Zhengzhou, China; ^2^ Department of Irradiation, The First Affiliated Hospital of Zhengzhou University, Zhengzhou, China

**Keywords:** nanoenzyme, radiotherapy, hypoxia, 125 I, esophageal cancer

## Abstract

Radioisotopes have long been leveraged for internal radiotherapy-mediated cancer treatment. However, such therapeutic approaches are associated with serious side effects, and their efficacy is limited by intratumoral hypoxia. Herein, we prepared a folic acid-decorated palladium decahedral platform capable of enhancing the radiotherapeutic efficacy of iodine-125 (^125^I) seed treatment. This decahedral nanoenzyme was able to target tumor regions and catalyze the conversion of intracellular H_2_O_2_ to O_2_, thereby alleviating hypoxia within the tumor microenvironment. In addition, palladium was hypoxia can be alleviated, on the other hand, palladium was able to enhance the radiotherapeutic energy deposition within tumor tissues. The results of this analysis indicated that synthesized decahedral constructs can efficiently target and modify the hypoxic tumor microenvironment while simultaneously enhancing radiation energy deposition therein. Relative to palladium nanodots, the prolonged *in vivo* circulation of these decahedral constructs better enabled them to facilitate sustained radiosensitization. Overall, the results of this study highlight a novel approach to improving the therapeutic utility of ^125^I seed interstitial implantation, thus underscoring an important direction for future clinical research.

## Introduction

Esophageal cancer is among the most common forms of human malignancy and is frequently characterized by progressive dysphagia (Sung et al., 2021). Radiotherapy (RT) is one of the primary treatments for esophageal cancer (Guo et al., 2021), but patients undergoing RT are highly susceptible to esophageal mucosal edema and radiation esophagitis, both of which can aggravate underlying dysphagia and result in serious eating disorders (Su et al., 2016). In addition to hampering the efficacy of RT treatment, this can also result in potentially life-threatening venous thrombosis.

RT is among the most common forms of cancer treatment, relying on localized irradiation as an antitumor modality. Different forms of *in vitro* accelerator therapy and *in vivo* radioisotope therapy can be classified into external beam radiotherapy (EBRT) and internal radioisotope therapy (RIT) strategies (Chao et al., 2018; Xie et al., 2019; Liu et al., 2020) Both EBRT and RIT rely on high-energy rays (such as X-rays or electron beams), particle beams (such as proton beams), or radioactive isotopes to indirectly or directly damage nuclear DNA *via* the induction of ionizing radiation, thereby inducing tumor cell apoptosis (Pei et al., 2021). While RT has emerged as a primary cancer treatment strategy, it is subject to significant limitations that constrain its utility. In order to kill tumor cells, EBRT must pass through the skin and proximal normal tissues without any tumor-specific targeting, resulting in damage, inflammation, and potential oncogenesis in these otherwise healthy tissues (Dai et al., 2017; Huang et al., 2021a). In contrast, RIT approaches rely upon the intratumoral delivery of radioactive sources using a needle or similar mechanism (Sau et al., 2017). These radioactive sources are typically classified according to the particle type which they emit in the form of ionizing radiation, including α, β, and Auger particle emitters (Maier-Hauff et al., 2011). ^255^Ac and ^213^Bi, which emit alpha particles, exhibit a limited penetration depth (28–100 μm) and are thus only suited to the treatment of small or residual tumors after surgery (Zhu et al., 2017). In contrast, β-emitting particles such as ^125^I and ^188^Re can penetrate deeper tissues (20–130 mm) while maintaining relatively low cytotoxicity (Yi et al., 2018). Certain radioisotopes (^123^I-123, ^125^I-125) achieve optimal therapeutic efficacy only when present within the nucleus, and they exhibit a very low penetration depth (<0.5 μm) (Pirovano et al., 2020; Shen et al., 2020). Relative to EBRT, RIT is better suited to tumor targeting, but as an invasive treatment it can nonetheless damage surrounding normal tissues (Song et al., 2017). The tumor microenvironment (TME) is a highly acidic and hypoxic compartment, and repeated EBRT or RIT can aggravate such hypoxia, resulting in hypoxic radiotherapy tolerance and further reducing RT efficacy (Finley and Popel, 2013; Satterlee et al., 2017). As such, the selection of an excessively high radiation dose that damages healthy tissues or an excessively low dose that fails to induce sufficient tumor cell death can lead to adverse outcomes. Appropriately balancing tissue protection and tumor cell killing remains a major challenge to the effective treatment of cancer patients (Ma et al., 2017).

Rapid advances in the development of nanotechnology have highlighted a range of strategies well-suited to enhancing the therapeutic efficacy of radionuclides. Several studies have indicated that specific nanomaterials can improve RIT sensitivity and associated treatment outcomes (Kwatra et al., 2013; Lomax et al., 2013; Mi et al., 2016), with known RIT sensitizing materials being broadly classified based upon the mechanisms whereby they mediate radiosensitization. Nanomaterials containing elements with a high atomic number can improve the therapeutic effect of RIT (Chao et al., 2018). As the photoelectric effect associated with ionizing radiation is directly proportional to the cubic power of an element’s atomic number, an increase in this photoelectric effect will thus enhance RIT radiotherapeutic efficacy. Le Goas et al. prepared a Hybrid Polymer (PMAA-AUNPs) *via* controlled free radical polymerization and determined that the resultant particles were able to increase the radiation dose deposited proximal to this polymer while significantly enhancing the cytotoxic effects of systemic ^125^I administration on tumor cells, thereby increasing RIT sensitivity (Le Goas et al., 2019). The targeting of radioactive sources can improve the killing of tumor cells while minimizing healthy tissue damage (Ge et al., 2020). Chen et al. synthesized a hybrid protein nanoreactor (^125^I-HSA-CAT), that, as it incorporated catalase, was able to catalyze the decomposition of endogenous H_2_O_2_ while simultaneously delivering ^125^I to the target tumors, thereby significantly enhancing the efficacy of RIT treatment (Finger and Giaccia, 2010; Chen et al., 2019). Modulating the TME thus represents an attractive approach to increasing the radiosensitivity of tumor cells, thereby maximizing radiation dose utilization. Tao et al. designed a composite nanomaterial (^125^I-rGO-MnO_2_-PEG) in which MnO_2_ was able to consume H_2_O_2_ within the TME to alleviate hypoxia, together with the systemic intravenous administration of ^125^I along with the nanocarrier-reducing nano-graphene oxide (rGO), enabling the synergistic enhancement of ^125^I-mediated radiotherapy *via* a thermotherapy approach (Tao et al., 2018). While these current internal radiation sensitizers can effectively improve the treatment effect of RIT, they are nonetheless subject to multiple shortcomings and limitations. For example, the rapid internal circulation *in vivo* can lead to the rapid metabolic processing of these sensitizers such that they are largely discharged or degraded before playing an effective therapeutic role (Semenza, 2016; Yi et al., 2016; Xia et al., 2020) Additionally, the complexities of living systems and the role of the immune system in humans and other animal models can prevent these sensitizers from achieving the desired therapeutic effects (Huang et al., 2021b).

To enhance the therapeutic efficiency of ^125^I, we herein developed a nanoenzyme platform capable of alleviating intratumoral hypoxia and thereby achieving robust radiosensitization. For this approach, we synthesized a decahedron consisting of palladium (Pd Decahedron), and these decahedral constructs were then decorated with folic acid to yield PD-FA. PD-FA was able to catalyze the conversion of high levels of intratumoral H_2_O_2_ into oxygen, thus reducing intratumoral hypoxia. Following PD-FA injection *via* the tail vein, it was able to target tumor regions in a folic acid-dependent manner. ^125^I was then injected intratumorally, releasing γ rays in tumors and thereby inducing indirect or direct DNA damage. The resultant DNA radicals can then be fixed by the generated oxygen. Overall, these PD-FA nanoparticles exhibit promising properties that make them efficient radiosensitizers in the context of ^125^I treatment.

## Results and Discussion

PD-FA nanoparticles were prepared *via* a two-step process wherein Pd decahedra were initially synthesized and were then coated with folic acid. Pd decahedra synthesis was conducted using a modified version of a previously published method. Briefly, 40 mg of Na_2_SO_4_ and 80 mg of polyvinyl pyrrolidone were dissolved and mixed in diethylene glycol at 105°C for 30 min, after which 15.5 mg of Na_2_PdCl_4_ was dissolved in 1 ml of diethylene glycol, and this solution was added to the prepared mixture. This solution was allowed to react for 5 h at 105°C, after which it was quenched with ice. Acetone was then added into the mixture, with was then centrifuged at 16,000 rpm to collect the product. This product was then washed twice with deionized water and dried overnight under vacuum. Folic acid surface modification was achieved by dispersing folic acid and Pd decahedra in absolute ethanol and stirring for 12 h under ultrasonication. The resultant product was then washed and dried under vacuum to yield PD-FA.

**FIGURE 1:**
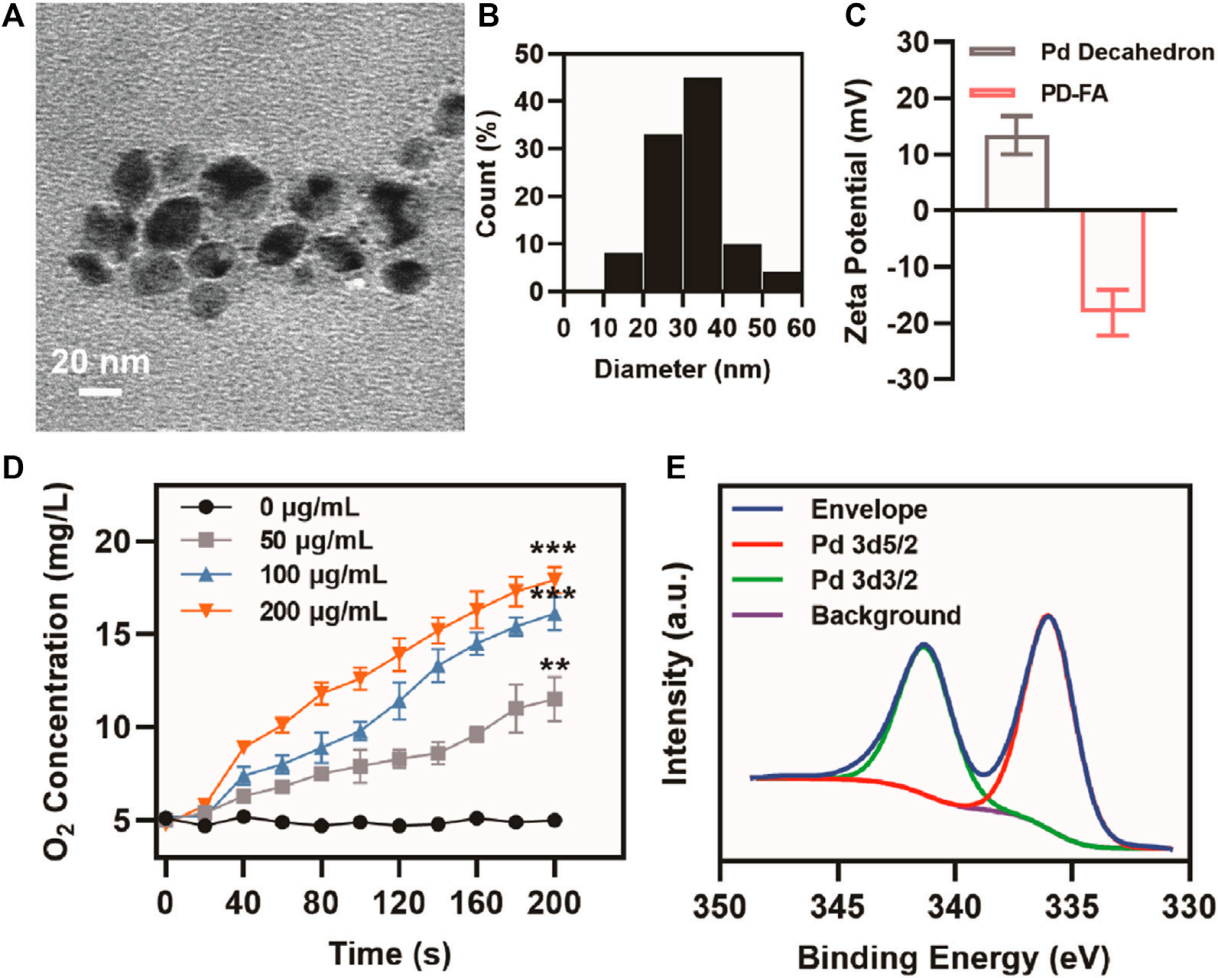
PD-FA characterization. **(A)** TEM images of PD-FA and **(B)** PD-FA size distributions. **(C)** Zeta potential values for Pd decahedra and PD-FA preparations. **(D)** Oxygen content measurements following the addition of H_2_O_2_ to PD-FA samples at the indicated concentrations. **(E)** XPS spectra corresponding to elemental Pd.

When Pd decahedra were analyzed *via* transmission electron microscopy (TEM), they exhibited a decahedral structure and were ∼30 nm in size (Supplementary Figure S1). PD-FA TEM images revealed the presence of a thin film surrounding the decahedron (Figure 1A). PD-FA size distribution profiles revealed these particles to have an average diameter of 31.9 nm (Figure 1B). Pd decahedra exhibited a zeta potential of 13.4 mV, but this shifted to −18.2 mV following folic acid addition, consistent with successful surface modification (Figure 1C). Various concentrations of PD-FA were then combined with H_2_O_2_. The X-ray photoelectron spectrum (XPS) for these preparations exhibited characteristic Pd 3d5/2 and Pd 3d3/2 bands at 341.1 and 336.0 eV, respectively (Figure 1E). The ability of PD-FA to catalyze H_2_O_2_ conversion into oxygen, confirming that at a PD-FA concentration of 200 μg/ml, the oxygen content reached 17.9 mg/L after 200 s (Figure 1D), with this being sufficient to alleviate hypoxia.

**FIGURE 2:**
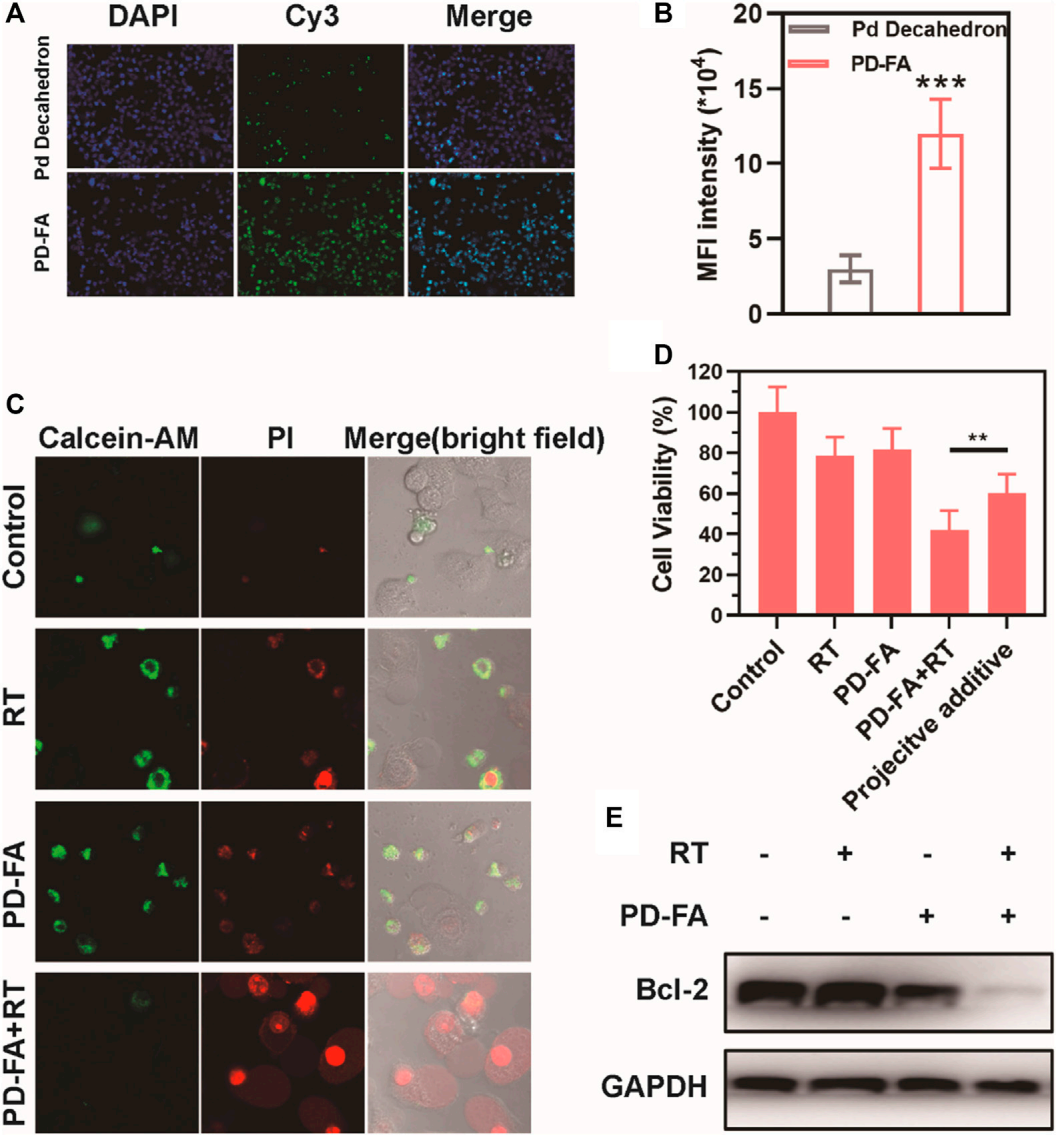
Assessment of the *in vitro* radiosensitization ability of PD-FA *in vitro*. **(A)** CLSM images of cells and **(B)** corresponding quantitative analyses of image fluorescence. **(C)** CLSM images of live and dead cells following the indicated treatments and **(D)** results of CCK-8 assay. **(E)** Western blotting results for cells in the indicated treatment groups.

Following successful PD-FA preparation, the ability of these nanoparticles to enhance radiosensitivity *in vitro* was assessed by using them to treat EC109 human esophageal cells. Both PD-FA and Pd decahedrons were labeled with Cy-5 and then cultured with EC109 cells, after which fluorescence was assessed *via* confocal laser scanning microscopy. Cells pretreated with PD-FA exhibited increased fluorescence intensity relative to those treated with Pd decahedrons (Figure 2A), consistent with enhanced PD-FA uptake owing to the targeting properties of folic acid. The median fluorescence intensity (MFI) of PD-FA-treated cells was 3.1-fold higher than that for Pd decahedron-treated cells (Figure 2B). In light of these promising results, we examined the radiosensitization activity of these cells by assessing EC109 cell apoptosis using Calcein-AM and propidium iodide (PI) to stain for live and dead cells, respectively. No significant cell death was evident in the RT or PD-FA treatment groups (Figure 2C), whereas in the presence of PD-FA, significant apoptosis was induced upon subsequent RT treatment. These results were further confirmed *via* a quantitative CCK-8 assay (Figure 2D), as RT or PD-FA treatments were associated with limited cellular apoptosis, whereas the viability of cells in the PD-FA + RT group decreased by 42.1%, consistent with the marked radiosensitization ability of PD-FA. B-cell lymphoma-2 (Bcl-2) is an important apoptosis-related gene, and Western blotting further demonstrated that Bcl-2 protein levels in the PD-FA + RT group were reduced relative to other treatment groups (Figure 2E), consistent with the increase in apoptotic cell death in this group.

**FIGURE 3:**
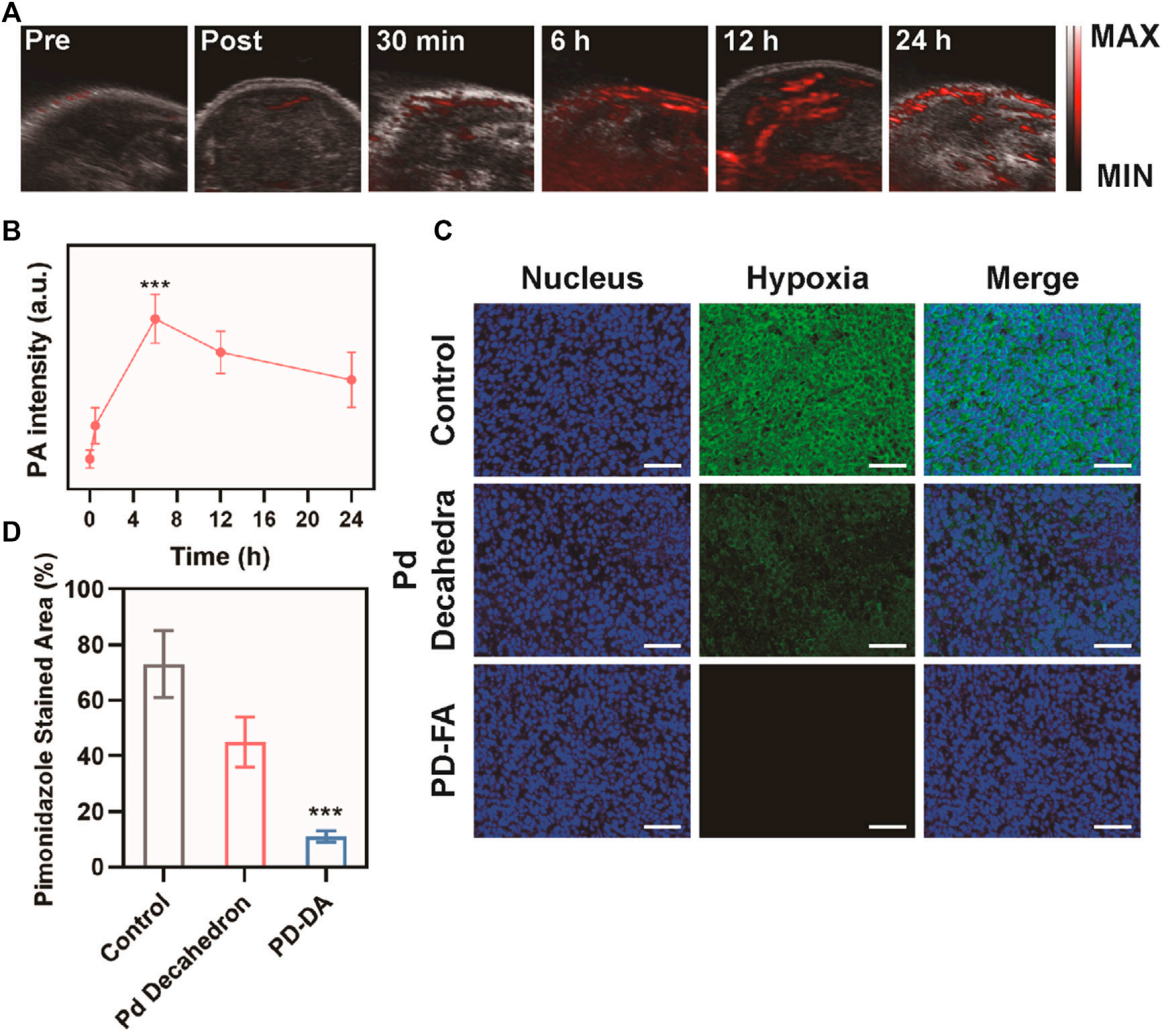
Assessment of the *in vivo* PD-FA-mediated alleviation of intratumoral hypoxia. **(A)** PA images of tumors pre-injection and at 30 min, 6, 12, and 24 h post-injection, with **(B)** corresponding PA signal intensity analysis. **(C)** immunofluorescence-stained tumor sections following the indicated treatments with **(D)** corresponding quantification of pimonidazole fluorescence (sacle bar: 50 μm).

A subcutaneous tumor model was next established to confirm the ability of PD-FA treatment to alleviate intratumoral hypoxia. PA imaging was utilized to measure *in vivo* oxygen content as a function of time in mice that were injected intravenously with PD-FA (Figures 3A,B). Following injection, oxygen levels in tumors rose until 6 h post-injection, and remained at relatively high levels until 24 h post-injection, consistent with the ability of PD-FA to successfully alleviate hypoxia. These results suggested that 6 h post-injection would be the optimal time to conduct RT treatment, given the maximal oxygen levels at this time. The tumor-targeting abilities of PD-FA and Pd decahedrons were additionally evaluated *via* immunofluorescence staining (Figure 3C). In the control group, tumors exhibited extensive hypoxia that was partially alleviated following Pd decahedron administration, consistent with the ability of these nanoparticles to increase oxygen levels. Importantly, PD-FA treatment significantly reduced hypoxia within these tumors (Figure 3D), with the pimonidazole stained area in control groups being 6.6 times that observed in the PD-FA group.

**FIGURE 4:**
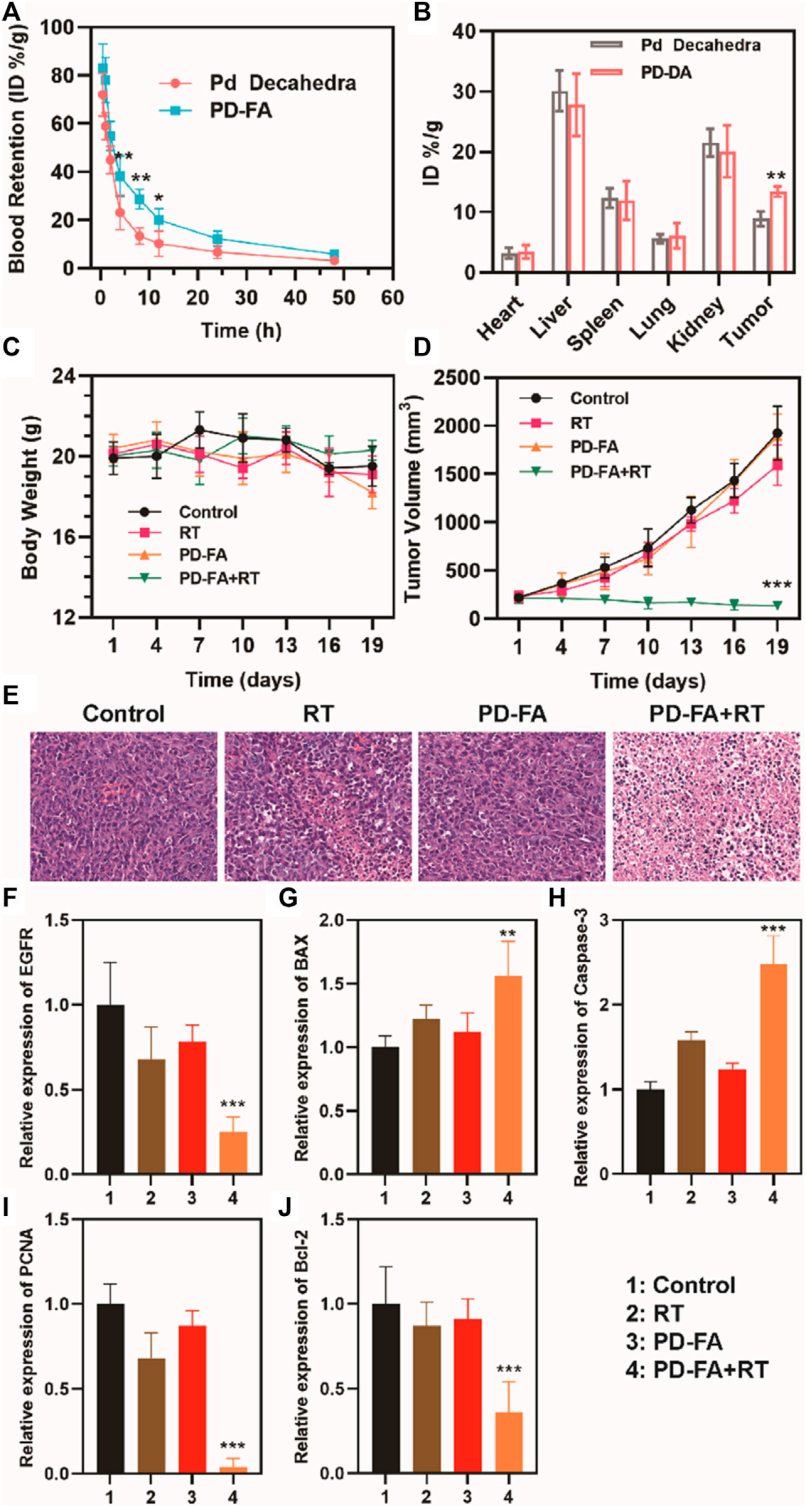
Analysis of antitumor efficacy. **(A)** Pharmacokinetic curves. **(B)** Biodistribution results for the indicated treatment groups. **(C)** Murine body weight and **(D)** tumor volumes in the four treatment groups. **(E)** HE staning of tumors in four groups. Quantitative analyses of the expression of **(F)** EGFR, **(G)** BAX, **(H)** Caspase-3, **(I)** PCNA, and **(J)** Bcl-2 in the indicated groups as assessed *via* RT-PCR.

In order to be clinically viable, the off-target toxicity of novel biomaterials must be assessed to ensure they exhibit satisfactory biocompatibility. To that end, we next intravenously injected nude BALB/c mice with PBS or a suspension of PD-FA in PBS. PD-FA injection was not associated with any apparent lesions in the major organs of injected mice (Supplementary Figure S2), suggesting that it does not induce any significant off-target cytotoxicity. We then assessed the antitumor efficacy of combination PD-FA + RT treatment by randomly assigning mice bearing EC109 tumors of a similar size into control and treatment groups that were intravenously injected with PBS or PD-FA and intratumorally injected with PBS or implanted with two radioactive ^125^I particles. Murine body weight and tumor volumes were monitored after treatment, revealing no obvious difference of body weight in all groups (Figure 4C) and sustained tumor growth in control animals only administered PBS (Figure 4D). In contrast, mice in the PD-FA + RT group exhibited the most extensive tumor ablation owing to the precise targeting activity and radiosensitization effects of PD-FA treatment. RT treatment alone was also sufficient to suppress tumor growth to some extent. On day 19, mice were euthanized and tumor tissues were collected. Subsequent hematoxylin and eosin (H&E) staining demonstrated extensive tumor cell apoptosis in the PD-FA + RT group (Figure 4E). Together, these data suggest that PD-FA + RT treatment can lead to near-complete tumor ablation. Corresponding pharmacokinetic curves (Figure 4A) indicated that these PD-FA nanoparticles are retained in circulation for extended periods owing to the utilized folic acid coating. Inductively coupled plasma-atomic emission spectrometry (ICP-AES) was used to assess Pd in tissue samples from these animals, revealing that PD-FA was associated with enhanced intratumoral accumulation and decreased hepatic and renal accumulation (Figure 4B). As such, these particles may achieve enhanced efficacy while reducing off-target toxicity. RT-PCR analyses further confirmed that HIF-1a and EGFR expression levels were reduced in the PD-FA + RT group relative to the control group (Figure 4F), while BAX and Caspase-3 expression exhibited the opposite phenotype (Figures 4G,H **)**. Decreased changes in Bcl-2 and PCNA expression were observed in the PD-FA + RT group (Figures 4I,J). Taken together, these results show that PD-FA + RT can induce marked intratumoral cellular apoptosis.

**SCHEME 1:**
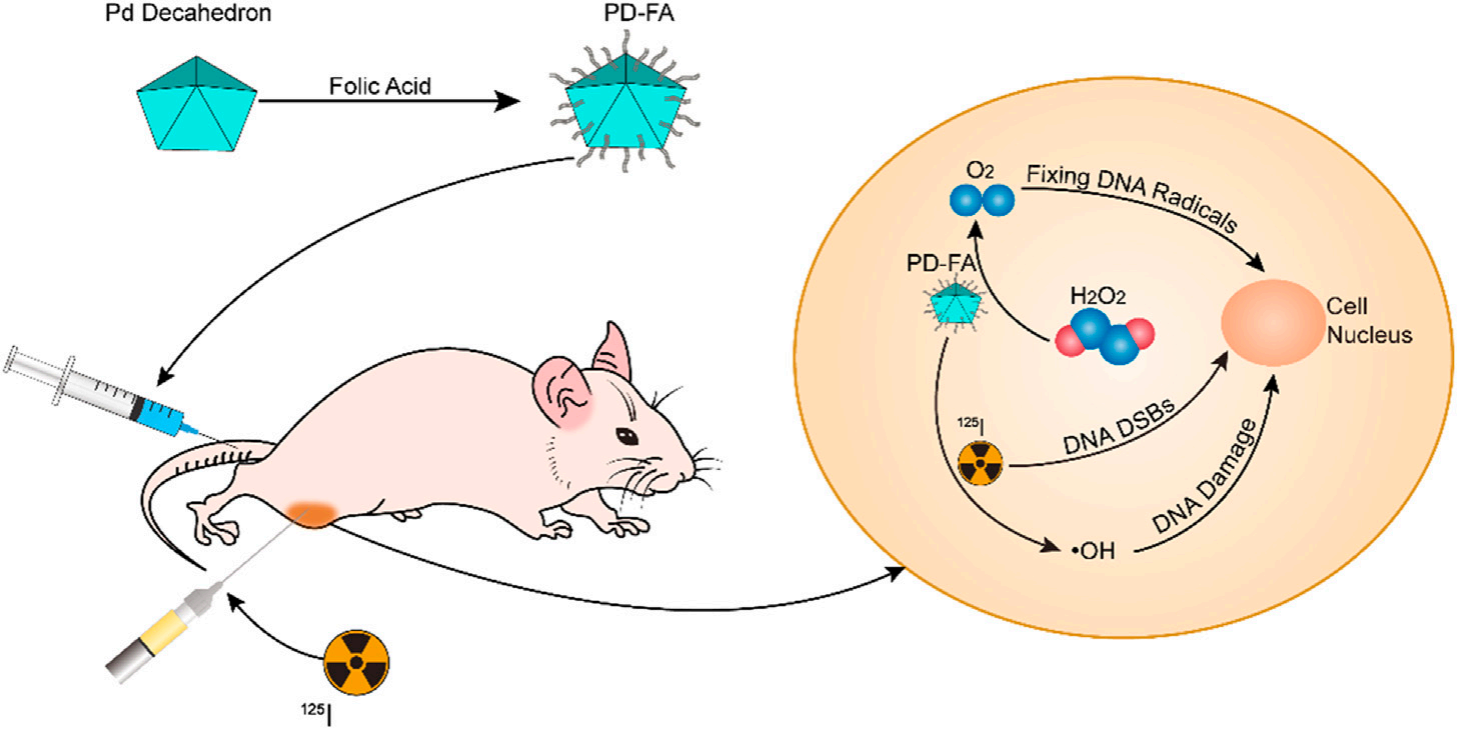
The synthesis of PD-FA and its application for *in vivo* radiosensitization.

## Conclusion

Hypoxia can impair the therapeutic efficacy of radiotherapy-based antitumor interventions. Herein, we successfully constructed a decahedral PD-FA nanoenzyme and leveraged it for the radiosensitization of RIT using ^125^I seed. PD-FA was able to efficiently and actively target tumor regions, therein catalyzing the conversion of H_2_O_2_ to oxygen and thus alleviating intratumoral hypoxia. IN addition, PD-FA was able to enhance ^125^I seed energy deposition within tumor regions, bolstering the generation of DNA radicals that can be fixed by oxygen cause permanent damage and induce cellular apoptosis. As such, this PD-FA nanoenzyme represents great potential for ^125^I seed radiosensitization, and exhibits good biocompatibility that warrants further clinical study.

## Materials and Methods

### Pd Decahedron and PD-FA Characterization

Pd decahedron and PD-FA NP morphological characteristics were assessed *via* transmission electron microscopy (TEM; Tecnai G2 F20 S-Twin, FEI, United States) at an acceleration voltage of 100 keV. Surface chemical elements were analyzed *via* XPS (ESCA-Lab250XI, Thermo Fisher Ltd., United States). The zeta potential and zeta-diameter measurements were quantified based upon dynamic light scattering (DLS, Nano-Zen 3,600, Malvern Instruments, United Kingdom).

### Oxygen Measurements

To assess the oxygen-generating capacity of PD-FA, we utilized a dissolved oxygen meter to measure oxygen concentrations under a nitrogen atmosphere to assess the impact of different PD-FA concentrations (0, 50, 100, 200 μg/ml) on H_2_O_2_ at concentration of 30 mM generation.

### Cell Culture

Human esophageal EC109 cancer cells (Chinese Academy of Sciences, Shanghai, China) were cultured in RPMI-1640 containing 10% FBS (Hyclone, United States) at 37°C in a humidified 5% CO_2_ incubator.

### *In Vitro* Cytotoxicity Analysis

A CCK-8 kit was used to assess *in vitro* EC109 cell killing. Initially, the impact of different PD-FA concentrations on cell survival following co-culture was assessed by plating EC109 cells overnight in 96-well plates (5,000/well), after which different PD-FA concentrations (0, 12.5, 25, 50, 100, 200 μg/ml) were added, and cells were incubated for an additional 24 h. Next, 10 μl of CCK-8 reagent was added per well, and plates were incubated for 2 h, after which a microplate meter (Rayto-6000 system, Rayto, China) was used to measure absorbance at 450 nm as a correlate for cytotoxicity. A CCK-9 kit was also used to assess cytotoxicity as a function of treatment type. Briefly, EC109 cells were plated and incubated overnight as above, after which they were separated into 4 treatment groups (5 wells/group): 1) Control; 2) PD-FA; 3) RT; and 4) PD-FA + RT. The PD-FA concentration for all groups was 50 μg/ml. Cells were treated as appropriate and incubated overnight, after which 10 μl of CCK-8 was added per well for 2 h, and absorbance was measured as above.

### Western Blotting

EC109 cells were separated into four treatment groups: 1) Control; 2) PD-FA; 3) RT; and 4) PD-FA + RT, with a PD-FA concentration of 50 μg/ml. Total protein was then isolated from treated cells, and Bcl-2 protein levels therein were assessed *via* Western blotting.

### Confirmation of *In Vitro* Cytotoxicity

To assess the cytotoxic effects of PD-FA treatment, EC109 cells were separated into four treatment groups: 1) control; 2) PD-FA; 3) RT; and 4) PD-FA + RT groups and were treated for 4 h in 60 mm tissue culture dishes. The PD-FA dose for these analyses was 50 μg/ml, while the RT dose was 22 MBq. Following the addition of FDA and PI, cells were rinsed using PBS and examined *via* fluorescence microscope (IX81, Olympus, Japan).

### Animal Model Establishment

Female BALB/c nude mice (4–5 weeks old, Vital River Company, Beijing, China) were subcutaneously injected with 1 × 10^5^ EC109 cells in a 100 μl volume on the right side of the abdomen. All the protocols for the procedures were approved by Animal Experiment Center of Wuhan University according to the protocols of the Institutional Animal Care and Use Committee (IACUC).

### Assessment of *In Vivo* Oxygenation

The *in vivo* oxygenation activity of PD-FA was assessed by removing the hair from tumor sites in five mice, and then scanning these sites with a Vevo Lazer system (Fujifilm, Visualsonics Inc. Canada) to obtain PA images of blood saturation. Next, 100 μl of PD-FA (200 μg/ml) was injected into the caudal vein, and the tumor site was scanned using this device at 0, 6, and 24 h.

For subsequent immunofluorescent analyses, mice in the control group and mice that had been injected with PD-FA 24 h previously were intraperitoneally injected with pimonidazole hydrochloride (60 mg/kg), after which tumors were collected and frozen. Tumor sections were then stained with anti-pimonidazole and HRP-conjugated rabbit anti-FITC (1:100), while blood vessels were stained with monoclonal rat anti-mouse CD31 (1:200) and a rhodamine-linked donkey anti-rat secondary antibody (1:200). DAPI (1:5,000) was used to stain cellular nuclei, and cells were imaged *via* confocal microscopy.

### Assessment of *In Vivo* Antitumor Efficacy

A total of 20 BALB/c nude mice were subcutaneously implanted on the right side of their abdomen with 100 μl of an EC109 cell suspension. When tumors were ∼100 mm^3^ in size, animals were randomized into four treatment groups (5 mice/group): 1) control; 2) PD-FA; 3) RT; and 4) PD-FA + RT groups. The PD-FA concentration for all animals was 200 μg/ml (100 μl) for once, and the RT dose was 22 MBq by implantation of two ^125^I particles following the injection. Tumor growth was monitored every 3 days using calipers, and changes in tumor volume and body weight were recorded.

### Analyses of Blood Biochemistry

When tumors had grown to 80–100 mm^3^ in size, mice were separated into three groups: 1) control mice, 2) mice injected with 100 μl of Pd decahedrons (200 μg/ml), and 3) mice injected with 100 μl of PD-FA (200 μg/ml) *via* the tail vein. Retroorbital blood samples were collected from these animals at 0.5, 2, 4, 8, 12, and 24 h, post-treatment.

### Safety Assessment of PD-FA

Healthy mice were divided into two groups to assess the *in vivo* biocompatibility of PD-FA. Mice of 6-week-old were intravenously administrated with PBS or PD-FA (100 μl; 200 μg/ml). 30 days post the injection, the mice were sacrificed and main organs including heart, liver, spleen, lung and kidney were harvest for further HE staning.

### RT-PCR Analysis of Tumor Tissues

After various treatment, mice in each group were sacrificed and tumor tissues were collected for RT-PCR to evaluate mRNA expression of EGFR, BAX, Caspase-3, PCNA, Bcl-2, respectively.

## Data Availability

The original contributions presented in the study are included in the article/Supplementary Material, further inquiries can be directed to the corresponding authors.
