# Supplementary material for: Development of A Decahedral Nanoenzyme Capable of Overcoming Hypoxia to Facilitate the Iodine-125 Radiosensitization of Esophageal Cancer
Source: Front Bioeng Biotechnol. 2021 Oct 8;9:764531. doi: 10.3389/fbioe.2021.764531 (PMC8531641; doi:10.3389/fbioe.2021.764531)
Supplement: Supplementary file 1 [file DataSheet1.docx]

*Supporting information for*

**Development of a decahedral nanoenzyme capable of overcoming hypoxia to facilitate the iodine-125 radiosensitization of** **esophageal cancer**

Dechao Jiao^1^, Kunpeng Wu^1^, Kaihao Xu^1^, Yiming Liu^1^, Deyao Zhao^2^, Xinwei Han^1^, Ruitai Fan^2^

Dechao Jiao and Kunpeng Wu shared the first co-author.

Department:

Dechao Jiao, Ph.D, Department of Interventional Radiology, The First Affiliated Hospital of Zhengzhou University, Zhengzhou, People’s Republic of China;

E-mail: jiaodechao007@126.com

Kunpeng Wu, MD, Department of Interventional Radiology, The First Affiliated Hospital of Zhengzhou University, Zhengzhou, 450052 Henan, People’s Republic of China; Department of Radiation and Medical Oncology, Hubei Key Laboratory of Tumor Biological Behaviors, Hubei Cancer Clinical Study Center, Zhongnan Hospital of Wuhan University, Wuhan, China, 430071

E-mail: wukunpeng0201@163.com

Kaihao Xu, MD, Department of Interventional Radiology, The First Affiliated Hospital of Zhengzhou University, Zhengzhou, 450052 Henan, People’s Republic of China;

E-mail: xukaihao7426@163.com

Yiming Liu, MD,Department of Interventional Radiology, The First Affiliated Hospital of Zhengzhou University, Zhengzhou, 450052 Henan, People’s Republic of China; E-mail: liuyiming1516@163.com

Deyao Zhao, Ph.D, Department of irradiation, The First Affiliated Hospital of Zhengzhou University, Zhengzhou, 450052 Henan, People’s Republic of China;

Email: zhaodeyao@hotmail.com

Xinwei Han, Ph.D, Department of Interventional Radiology, The First Affiliated Hospital of Zhengzhou University, Zhengzhou, 450052 Henan, People’s Republic of China;

E-mail: 13592583911@163.com

Ruitai Fan, Ph.D, Department of irradiation, The First Affiliated Hospital of Zhengzhou University, Zhengzhou, 450052 Henan, People’s Republic of China; E-mail: fccfanrt@zzu.edu.cn

Information of Corresponding Author:

Prof. Xinwei Han and Ruitai Fan

Xinwei Han, Ph.D

Department of Interventional Radiology, The First Affiliated Hospital of Zhengzhou University, Zhengzhou, Henan, People’s Republic of China;

Tel: +0086-13803842129

Email: 13592583911@163.com

Ruitai Fan, Ph.D

Department of irradiation, The First Affiliated Hospital of Zhengzhou University, Zhengzhou, 450052 Henan, People’s Republic of China;

E-mail: fccfanrt@zzu.edu.cn


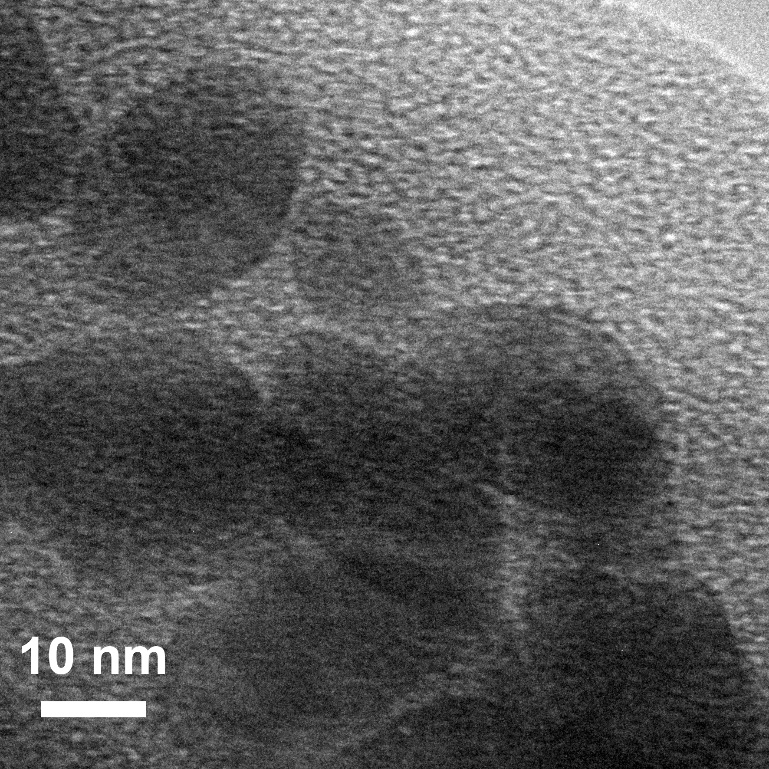


**Figure S1** TEM images of Pd Decahedron.


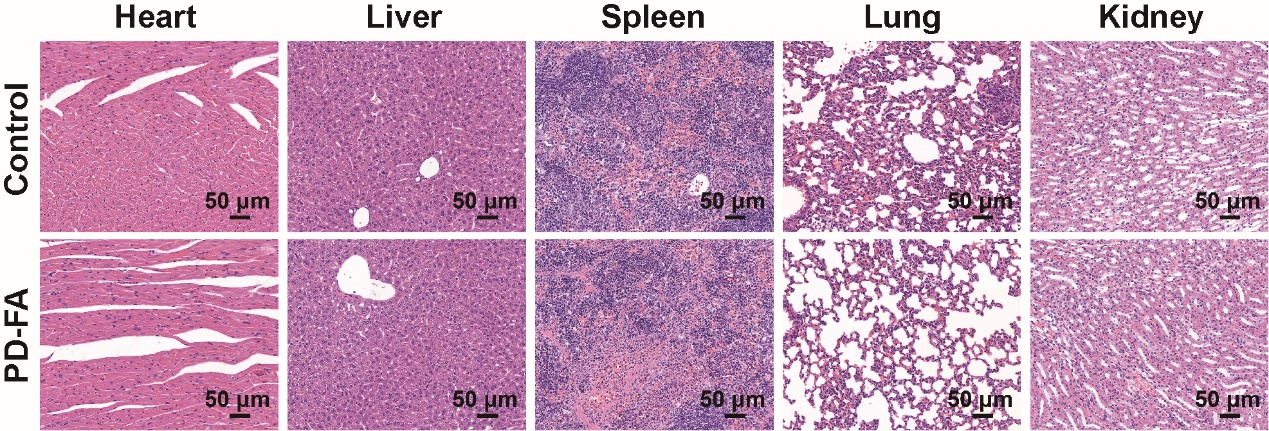


**Figure S2** HE staining of main organs.
